# Influence of exogenous ascorbic acid and glutathione priming on mitochondrial structural and functional systems to alleviate aging damage in oat seeds

**DOI:** 10.1186/s12870-020-2321-x

**Published:** 2020-03-06

**Authors:** Fangshan Xia, Hang Cheng, Lingling Chen, Huisen Zhu, Peisheng Mao, Mingya Wang

**Affiliations:** 1 College of Animal Science and Veterinary Medicine, Shanxi Agricultural University, Taigu, 030801 China; 2 Forage Seed Laboratory/Beijing Key Laboratory of Grassland Science, China Agricultural University, No 2, Yuanmingyuan West Road, Haidian Distr, Beijing, 100193 China

**Keywords:** Ascorbic acid-glutathione cycle, Mitochondria, Oat, Seed aging, Seed priming

## Abstract

**Background:**

Loss of vigor caused by seed aging adversely affects agricultural production under natural conditions. However, priming is an economical and effective method for improving the vigor of aged seeds. The objective of this study was to test the effectiveness of exogenous ascorbic acid (ASC) and glutathione (GSH) priming in the repairing of aged oat (*Avena sativa*) seeds, and to test the hypothesis that structural and functional systems in mitochondria were involved in this process.

**Results:**

Oat seeds were artificially aged for 20 days at 45 °C, and were primed with solutions (1 mmol L^− 1^) of ASC, GSH, or ASC + GSH at 20 °C for 0.5 h before or after their aging. Seed germination, antioxidant enzymes in the ASC-GSH cycle, cytochrome c oxidase (COX) and mitochondrial malate dehydrogenase (MDH) activities, and the mitochondrial ultrastructures of the embryonic root cells were markedly improved in aged oat seeds through post-priming with ASC, GSH, or ASC + GSH, while their malondialdehyde and H_2_O_2_ contents decreased significantly (*P* < 0.05).

**Conclusion:**

Our results suggested that priming with ASC, GSH, or ASC + GSH after aging could effectively alleviate aging damage in oat seeds, and that the role of ASC was more effective than GSH, but positive effects of post-priming with ASC and GSH were not superior to post-priming with ASC in repairing aging damage of aged oat seeds. However, pre-priming with ASC, GSH, or ASC + GSH was not effective in oat seeds, suggesting that pre-priming with ASC, GSH, or ASC + GSH could not inhibit the occurrence of aging damage in oat seeds.

## Background

Seed aging, resulting in a loss of vigor and viability under natural storage conditions, is a major problem for successful agricultural production and productivity. Moreover, seed aging also leads to ecological problems due to shortages of genetic resources and soil seed bank systems dysfunction, e.g., loss of plant biodiversity, grassland degeneration, and the enhancement of desertification [1]. Thus, loss of seed vigor is a major challenge for food and ecological security under natural conditions [2]. As a result, it is necessary to better understand the possible methods and mechanisms of maintaining seed vigor.

Accumulation of reactive oxygen species (ROS) is a primary cause of seed aging during storage, causing lipid peroxidation, impairment of RNA and protein synthesis, degradation of DNA, and loss of membrane integrity [3, 4]. The levels of ROS are tightly controlled by enzymatic and non-enzymatic antioxidant systems during seed aging [1]. Once ^·^O_2_^−^ is generated by the electron transport chain during seed aging, it is promptly dismutated into hydrogen peroxide (H_2_O_2_) by superoxide dismutase (SOD) in the matrix [5, 6]. H_2_O_2_ is then eliminated via the various antioxidant pathways that are crucial for the production and scavenging of ROS [7]. In particular, the scavenging of ROS largely depends on the availability of molecular antioxidants such as ascorbic acid (ASC) and glutathione (GSH) in dry seeds [8]. ASC and GSH are necessary to maintain a net reducing environment by reacting with ROS or participating in the ASC-GSH cycle [9, 10]. Importantly, the ASC-GSH cycle is a crucial detoxifying mechanism in both dry and imbibed seeds, and is principally located in embryos [11]. In the ASC-GSH cycle, ascorbate peroxidase (APX) reduces the toxicity of ROS by using ASC as substrate; ASC is then recycled in the matrix by NADH-dependent monodehydroascorbate reductase (MDHAR) or GSH-dependent dehydroascorbate reductase (DHAR), while GSH can be recycled in the matrix by glutathione reductase (GR) using NADPH as an electron donor [12, 13]. A decline in cellular ASC indicates failing antioxidant capacity during seed aging, hence contributing to a loss of seed viability [14–16]. Similarly, cellular GSH also declines rapidly, with a concomitant rapid decrease in seed vigor, during aging [4, 17, 18]. At the cellular level, seed aging also reduces the activities of several enzymes involved in the ASC-GSH cycle [16, 19]. Therefore, maintaining high levels of ASC and GSH is important to guarantee vigor in aged seeds.

Mitochondria customarily play a central role in plant metabolism as a major source of adenosine triphosphate (ATP) [20, 21]. Seeds are largely dependent on mitochondrial respiration to provide obligatory energy for their germination [22]. However, the activity of almost all enzymes involved in the tricarboxylic acid cycle decreases significantly during seed aging, e.g., the aging of elm (*Ulmus pumila*) [23] and rice (*Oryza sativa*) [24] seeds. Mitochondrial ultrastructure is impaired along with a significant decrease in the activities of cytochrome c oxidase (COX) and malate dehydrogenase (MDH), thereby creating an insufficient supply of mitochondrial energy and a surge in ROS [24]. Consequently, mitochondria play a dominant role in the generation of ROS in seeds [25], and ROS-related mitochondrial dysfunctions play a pivotal role in seed aging [23]. Similar to cellular antioxidant systems, the ASC-GSH cycle is also one of the major antioxidant protection systems in mitochondria under oxidative stresses [10, 26]. A distinct reduction has been observed in the content of both ASC and GSH and the activities of ASC-GSH cycle enzymes during seed aging [7, 25]. Nevertheless, there is still only a limited understanding of how to enhance mitochondrial function by improving the antioxidant capacity of the ASC-GSH cycle.

Seed priming can enhance the vigor of aged seeds by efficaciously repairing cellular and mitochondrial components [27, 28]. The mechanism of priming-enhanced seed longevity is likely associated with antioxidants [29, 30]. Consequently, priming with exogenous antioxidants, e.g., ASC and GSH, may be an important method for effectively improving the vigor of aged seeds. Studies have shown that exogenous ASC clearly promotes the germination of aged seeds in many plants, such as onion (*Allium cepa*) [31] and *Elymus sibiricus* [32, 33]. Nonetheless, previous studies rarely focused on the mechanism of exogenous ASC in promoting the germination of aged seeds, particularly at the mitochondrial level. Moreover, exogenous GSH pre-treatment can also improve the germination and antioxidant ability of aged *Elymus sibiricus* seeds [34], but there are still only a few studies on the role of exogenous GSH in improving the vigor of aged seeds. Therefore, it is necessary to better understand the effect of exogenous ASC and GSH on mitochondrial structures and functions of aged seeds in order to more effectively maintain their vigor.

Oat (*Avena sativa*), an eco-friendly crop, is widely cultivated throughout the word because it can adapt to a variety of environmental stresses. However, oat grains often have a high lipid content that can easily lead to rancidity or deterioration, hence limits its extensive use as a seed or food [35, 36]. Therefore, the objectives of this study were to determine the changes in the repair of ultrastructural structures, the enhancement of antioxidant capacity, the remission of lipid peroxidation, the recovery of respiratory functions in embryonic mitochondria of aged oat seeds, and to better understand the response of priming with exogenous ASC and GSH on the mitochondria in aged seeds.

## Results

### Effect of ASC and GSH priming on the germination percentage in aged oat seeds

**Fig. 1:**
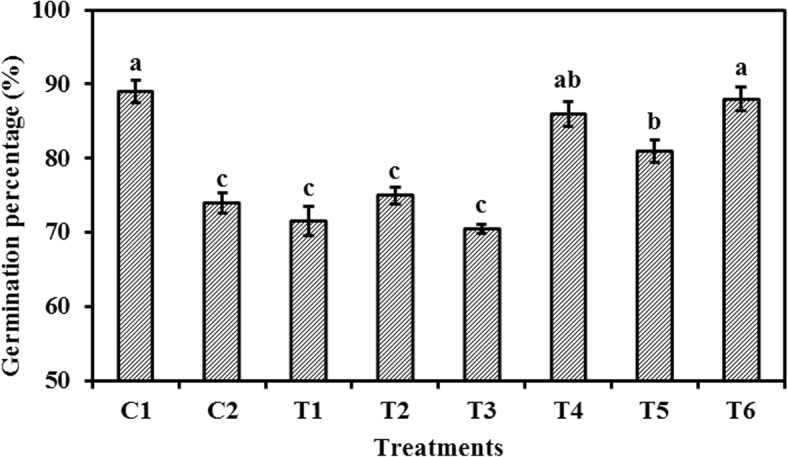
Effect of ASC or GSH priming on germination percentage of aged oat seeds. C1 unaged, unprimed oat seeds. C2 aged, unprimed oat seeds. T1 oat seeds primed with ASC before aging. T2 oat seeds primed with GSH before aging. T3 oat seeds primed with ASC + GSH before aging. T4 oat seeds primed with ASC after aging. T5 oat seeds primed with GSH after aging. T6 oat seeds primed with ASC + GSH after aging. Means with different letters are significantly different at the 0.05 level among treatments. Vertical bars represent the means of four independent determinations ±SE

The germination percentages of oat seeds primed with ASC or GSH after aging were higher than those primed before aging (Fig. 1). There were no significant differences (*P* > 0.05) in the germination percentages between oat seeds primed with ASC, GSH, or ASC + GSH before aging and those aged and non-primed. However, the germination percentages of oat seeds primed with ASC, GSH, or ASC + GSH after aging were significantly (*P* < 0.05) higher than those aged and non-primed. The germination percentage of oat seeds primed with ASC + GSH after aging was significantly (P < 0.05) higher than those primed with GSH after aging, but the germination percentages of oat seeds primed with ASC or ASC + GSH after aging were not significantly (*P* > 0.05) different from those unaged and non-primed.

### Effect of ASC and GSH priming on the mitochondrial structure of embryonic root cells in aged oat seeds

**Fig. 2:**
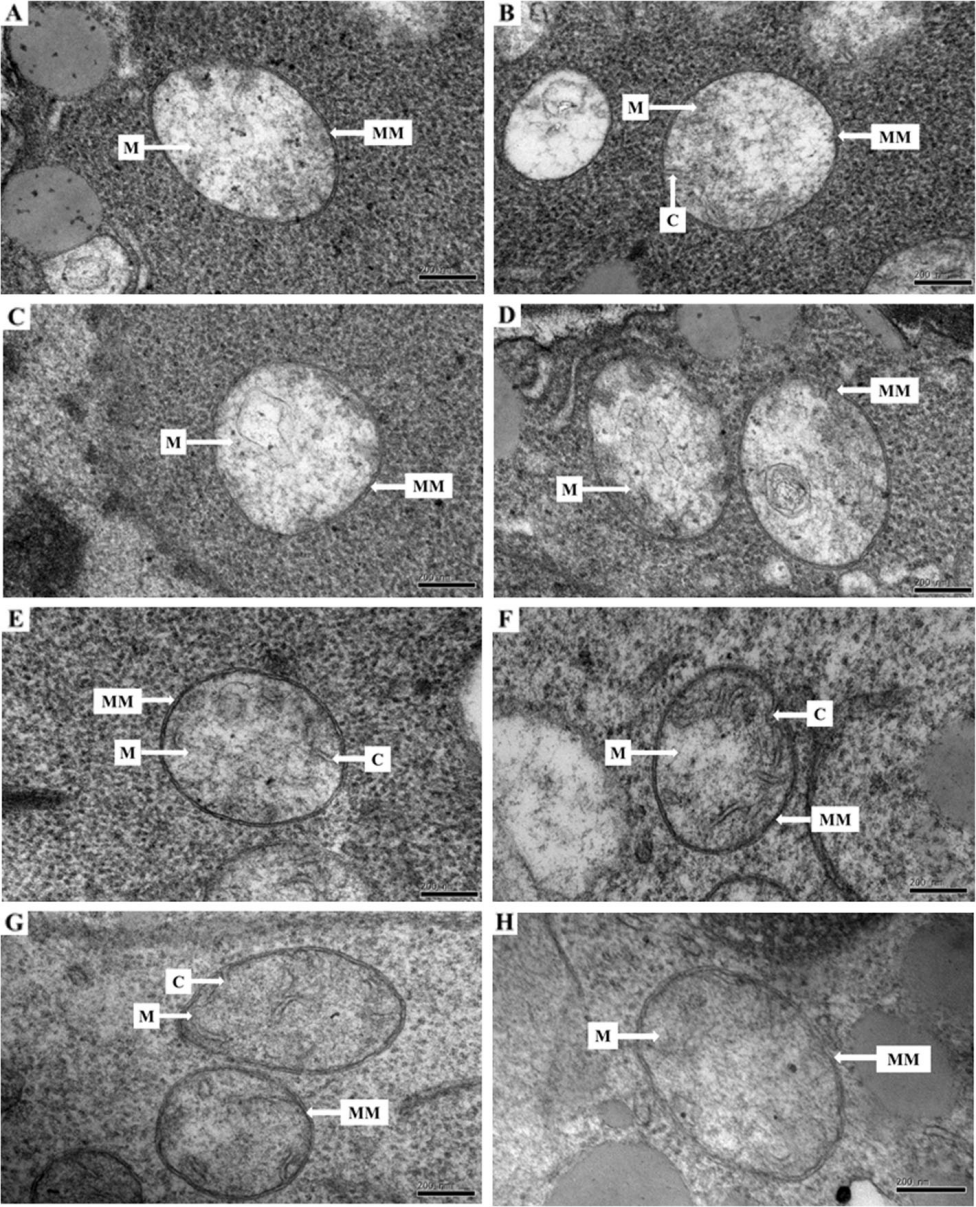
Effect of ASC or GSH priming on mitochondrial structures of embryonic root cells in aged oat seeds. **a** oat seeds primed with ASC before aging. **b** oat seeds primed with GSH before aging. **c** oat seeds primed with ASC + GSH before aging. **d** aged, unprimed oat seeds. **e** oat seeds primed with ASC after aging. F oat seeds primed with GSH after aging. **g** oat seeds primed with ASC + GSH after aging. H unaged, unprimed oat seeds. Bars = 200 nm. C cristae. M mitochondria. MM mitochondrial membrane

Ultrastructural observation of embryonic root cells showed that the mitochondrial structures of oat seeds primed with ASC, GSH, or ASC + GSH after aging were superior to those primed before aging (Fig. 2). The mitochondrial structures of embryonic root cells were imperfect in oat seeds primed with ASC or ASC + GSH before aging, and their inner mitochondrial membranes and cristae were indistinct (Fig. 2a and c). The mitochondrial membranes of embryonic root cells were clearly visible in both oat seeds non-primed (aged or unaged) and those primed with GSH before aging. However, their cristae were also indistinct (Fig. 2b, d, and h). Conversely, the mitochondrial membranes and cristae of embryonic root cells were clearly visible in oat seeds primed with ASC or ASC + GSH after aging (Fig. 2e and g). The mitochondrial structures of embryonic root cells were also intact in oat seeds primed with GSH after aging, but their double membranes were indistinct (Fig. 2f).

### Effect of ASC and GSH priming on mitochondrial enzymatic activity in embryo cells of aged oat seeds

**Fig. 3:**
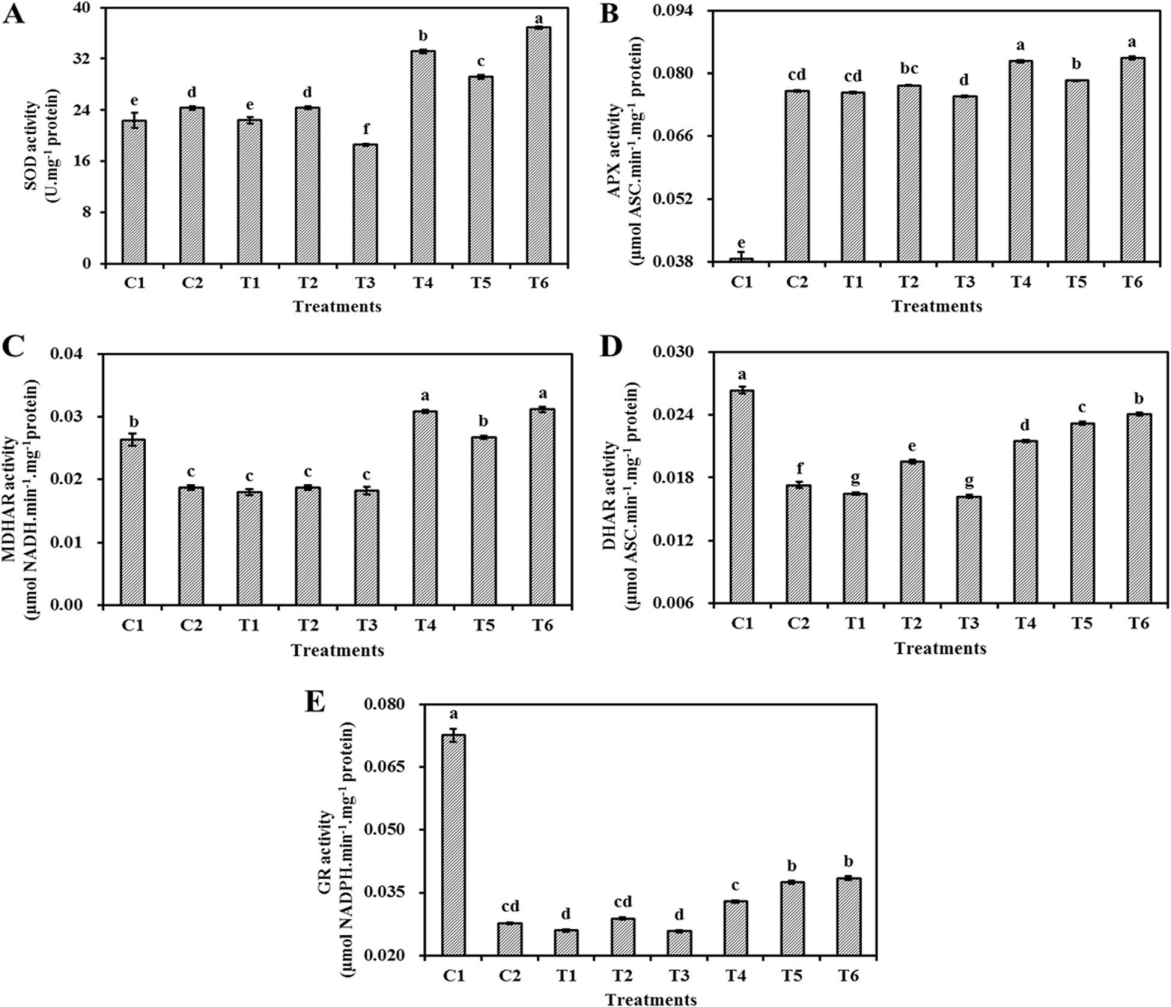
Effect of ASC or GSH priming on mitochondrial antioxidant enzymes of embryo cells in aged oat seeds. **a** SOD activity. **b** APX activity. **c** MDHAR activity. **d** DHAR activity. **e** GR activity. C1 unaged, unprimed oat seeds. C2 aged, unprimed oat seeds. T1 oat seeds primed with ASC before aging. T2 oat seeds primed with GSH before aging. T3 oat seeds primed with ASC + GSH before aging. T4 oat seeds primed with ASC after aging. T5 oat seeds primed with GSH after aging. T6 oat seeds primed with ASC + GSH after aging. Means with the different letters are significantly different at the 0.05 level among treatments. Vertical bars represent means of four independent determinations ±SE

The mitochondrial SOD, APX, MDHAR, DHAR, and GR activities in oat seeds primed with ASC, GSH, or ASC + GSH after aging were significantly (*P* < 0.05) higher than in those primed before aging (Fig. 3).

Mitochondrial SOD activities of oat seeds primed with ASC + GSH before aging were significantly (*P* < 0.05) lower than the others, but mitochondrial SOD activities of oat seeds primed with ASC + GSH after aging were significantly (P < 0.05) higher than the others (Fig. 3a). Mitochondrial SOD activities of aged, non-primed oat seeds were not significantly (*P* > 0.05) different from those primed with GSH before aging. However, mitochondrial SOD activities of non-primed oat seeds (aged or unaged) were significantly (*P* < 0.05) lower than those primed with GSH after aging.

Mitochondrial APX activities of unaged, non-primed oat seeds were significantly (P < 0.05) lower than the others (Fig. 3b). Mitochondrial APX activities of oat seeds primed before aging were not significantly (*P* > 0.05) different from those aged and non-primed, but mitochondrial APX activities of oat seeds primed with ASC or ASC + GSH after aging were significantly (*P* < 0.05) higher than the others. Mitochondrial APX activities of aged and non-primed oat seeds were significantly (P < 0.05) lower than those primed with GSH after aging.

Mitochondrial MDHAR activities of aged, non-primed oat seeds were not significantly (*P* > 0.05) different from those primed before aging (Fig. 3c). However, mitochondrial MDHAR activities of oat seeds primed with ASC, GSH, or ASC + GSH after aging were significantly higher (*P* < 0.05) than those primed before aging. The highest level of mitochondrial MDHAR activities was found in oat seeds primed with ASC or ASC + GSH after aging. There were no significant (P > 0.05) differences in mitochondrial MDHAR activities between unaged, non-primed oat seeds and those primed with GSH after aging.

Mitochondrial DHAR activities were highest in unaged, non-primed oat seeds, but were lowest in those primed with ASC or ASC + GSH before aging, and there were no significant (P > 0.05) differences between those primed with ASC and ASC + GSH before aging (Fig. 3d). However, the mitochondrial DHAR activities of oat seeds primed after aging were significantly (*P* < 0.05) higher than those aged and non-primed. Mitochondrial DHAR activities of oat seeds primed with ASC + GSH after aging were significantly (*P* < 0.05) higher than other aged oat seeds. Additionally, mitochondrial DHAR activities were significantly (*P* < 0.05) higher in oat seeds primed with GSH either before or after aging than in those aged and non-primed.

Mitochondrial GR activities were highest in unaged, non-primed oat seeds, but were lowest in those primed with ASC or ASC + GSH before aging (Fig. 3e). However, mitochondrial GR activities of aged, non-primed oat seeds were significantly (*P* < 0.05) lower than those primed with GSH or ASC + GSH after aging.

### Effect of ASC and GSH priming on mitochondrial H_2_O_2_ and MDA content in embryo cells of aged oat seeds

**Fig. 4:**
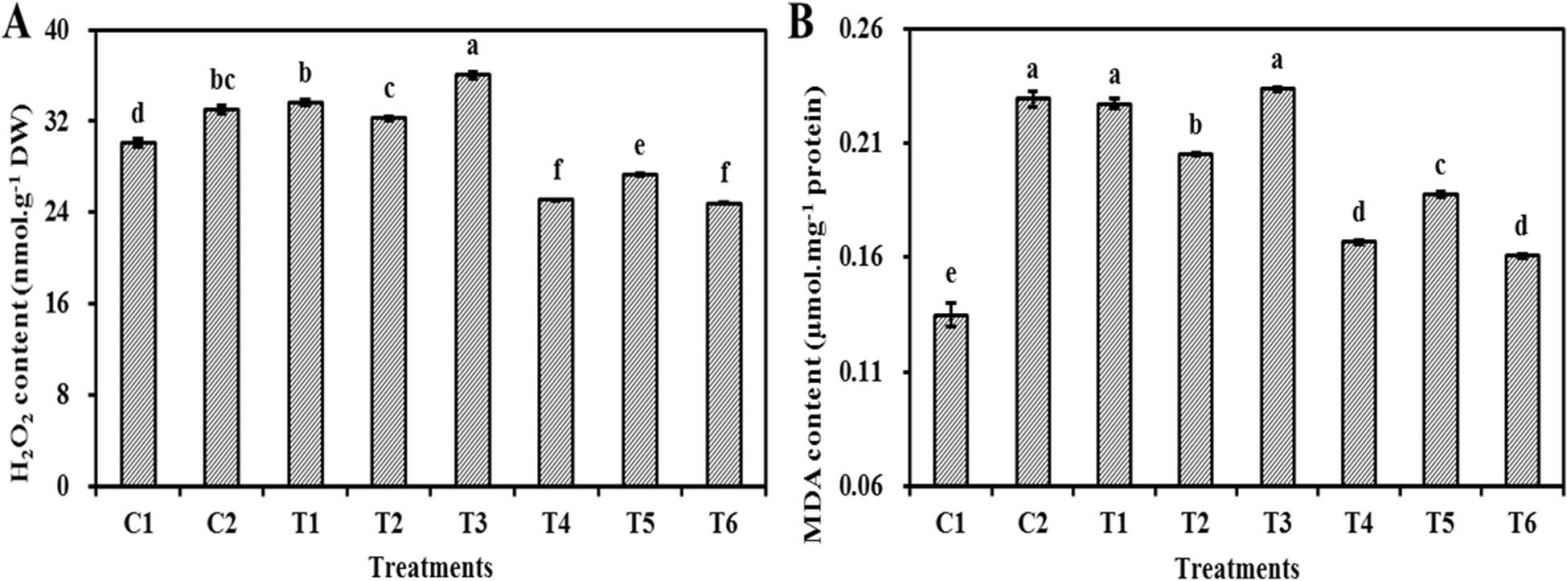
Effect of ASC or GSH priming on mitochondrial H_2_O_2_ and MDA contents of embryo cells in aged oat seeds. **a** H_2_O_2_ content. **b** MDA content. C1 unaged, unprimed oat seeds. C2 aged, unprimed oat seeds. T1 oat seeds primed with ASC before aging. T2 oat seeds primed with GSH before aging. T3 oat seeds primed with ASC + GSH before aging. T4 oat seeds primed with ASC after aging. T5 oat seeds primed with GSH after aging. T6 oat seeds primed with ASC + GSH after aging. Means with the different letters are significantly different at the 0.05 level among treatments. Vertical bars represent means of four independent determinations ±SE

Mitochondrial H_2_O_2_ contents of unaged, non-primed oat seeds were significantly (*P* < 0.05) higher than those primed with ASC, GSH, or ASC + GSH after aging, but they were significantly (P < 0.05) lower than those primed before aging and those aged and non-primed (Fig. 4a). Mitochondrial H_2_O_2_ contents were highest in oat seeds primed with ASC + GSH before aging, but they were significantly (*P* < 0.05) lower in oat seeds primed with GSH before aging than those primed with ASC before aging. Mitochondrial MDA contents of unaged, non-primed oat seeds were significantly (*P* < 0.05) lower than the others (Fig. 4b). Mitochondrial MDA contents of oat seeds primed with GSH before aging were significantly (*P* < 0.05) lower than those aged and non-primed, but mitochondrial MDA contents of oat seeds primed with ASC or ASC + GSH before aging were not different from those aged and non-primed. Mitochondrial H_2_O_2_ and MDA contents in oat seeds primed with ASC, GSH, or ASC + GSH after aging were significantly (*P* < 0.05) lower than those aged and non-primed, and the lowest level was observed in those primed with ASC or ASC + GSH after aging.

### Effect of ASC and GSH priming on mitochondrial COX and MDH activities in embryo cells of aged oat seeds

**Fig. 5:**
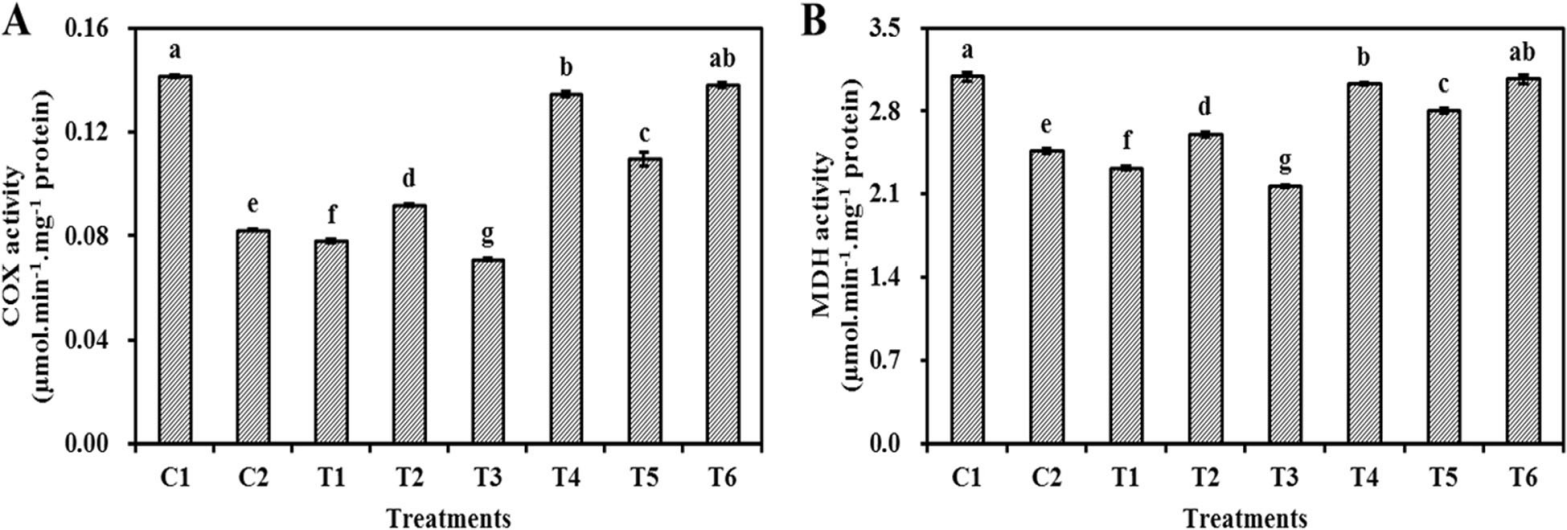
Effect of ASC or GSH priming on mitochondrial COX and MDH activity of embryo cells in aged oat seeds. **a** COX activity. **b** MDH activity. C1 unaged, unprimed oat seeds. C2 aged, unprimed oat seeds. T1 oat seeds primed with ASC before aging. T2 oat seeds primed with GSH before aging. T3 oat seeds primed with ASC + GSH before aging. T4 oat seeds primed with ASC after aging. T5 oat seeds primed with GSH after aging. T6 oat seeds primed with ASC + GSH after aging. Means with the different letters are significantly different at the 0.05 level among treatments. Vertical bars represent means of four independent determinations ±SE

The changes in mitochondrial COX and MDH activities were similar, and the activities of mitochondrial COX and MDH activities were significantly (P < 0.05) higher in oat seeds primed with ASC, GSH, or ASC + GSH after aging than in those primed before aging (Fig. 5). Mitochondrial COX and MDH activities were significantly (P < 0.05) lower in oat seeds primed with ASC or ASC + GSH before aging than in those aged and non-primed, and the lowest level was found in those primed with ASC + GSH before aging. However, mitochondrial COX and MDH activities were significantly (P < 0.05) higher in oat seeds primed with GSH before aging than in those aged and non-primed. Mitochondrial COX and MDH activities maintained a high level in oat seeds primed with ASC or ASC + GSH after aging and in those unaged and non-primed.

## Discussion

**Fig. 6:**
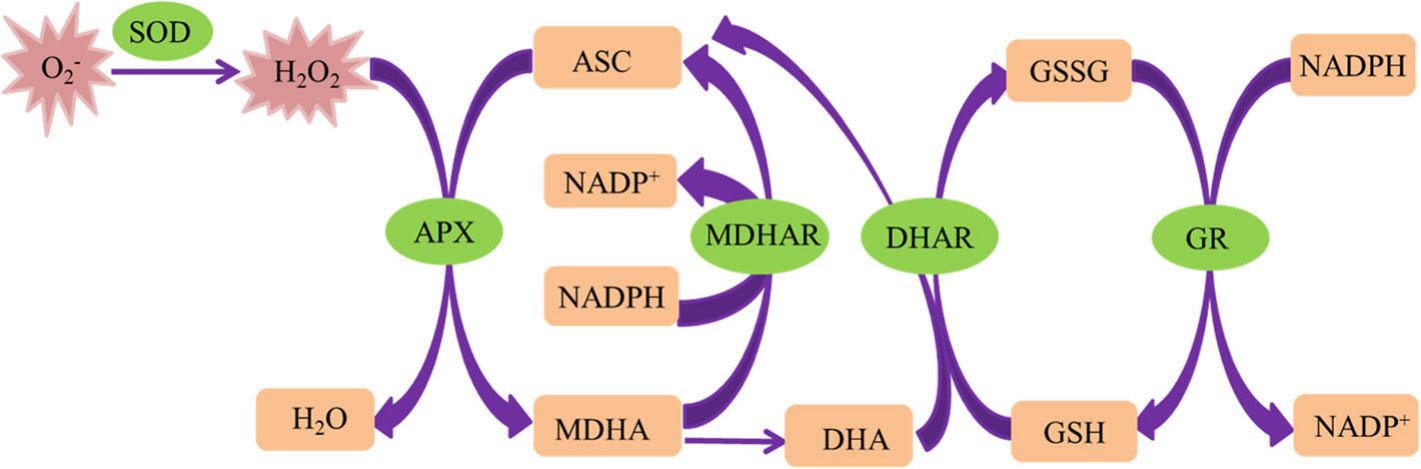
The ASC-GSH cycle in mitochondrion of plant [38]. SOD superoxide dismutase. H_2_O_2_ hydrogen peroxide. APX ascorbate peroxidase. ASC ascorbic acid. MDHA monodehydroascorbic acid. MDHAR monodehydroascorbic acid reductase. DHA dehydroascorbic acid. DHAR dehydroascorbic acid reductase. GSH glutathione. GSSG oxidized glutathione. GR glutathione reductase

Fully functional mitochondria often have extensive cristae structures and various biochemical activities [37], and the ASC-GSH cycle is one of the primary antioxidant protection systems in mitochondria under various oxidative stresses (Fig. 6) [39]. However, mitochondrial ultrastructural damage in response to artificial aging negatively impacts mitochondrial function [25]. Previous research also suggests that a decrease in the activities of antioxidant enzymes participating in the ASC-GSH cycle, accompanied by mitochondrial ultrastructural damage, is the major cause of aging in oat seeds [7]. Our current results showed that the negative influence of aging in oat seeds could be effectively repaired by post-priming with ASC, GSH, or ASC + GSH (Fig. 3). Compared to aged and non-primed oat seeds, the mitochondrial SOD, APX, MDHAR, DHAR, and GR activities were all markedly increased in those primed with ASC, GSH, or ASC + GSH after aging. Furthermore, the activities of mitochondrial SOD and APX were almost always higher in oat seeds primed with ASC, GSH, or ASC + GSH after aging than in those unaged and non-primed (Fig. 3). These results indicated that priming after aging prevented the depression in antioxidant ability of ASC-GSH cycle’ enzymes in oat seeds and was therefore beneficial in supporting the removal of ROS using ASC and GSH. Similarly, priming not only activates existing enzymes, but also improves rRNA integrity, leading to higher levels of protein synthesis in seeds [3]. Consequently, mitochondrial H_2_O_2_ and MDA contents of oat seeds primed with ASC, GSH, or ASC + GSH after aging were also lower than those aged and non-primed (Fig. 4). These results suggested that lipid peroxidation could be effectively relieved in oat seeds by priming with ASC, GSH, or ASC + GSH after aging. Mitochondrial structures of embryonic root cells were obviously restored as cristae gradually emerged in oat seeds primed with ASC, GSH, or ASC + GSH after aging (Fig. 2d-g). This was consistent with our previous findings, which showed that the integrity of the mitochondrial membrane was related to the levels of mitochondrial antioxidants and MDA contents in aged oat seeds [7]. ATP production in mitochondria is the main source of energy during seed germination [25], but oxidative modification can reduce the activities of COX and MDH during seed aging, leading to a decrease in the ATP production [23, 24]. In this study, the activities of mitochondrial COX and MDH maintained a high level in oat seeds primed with ASC, GSH, or ASC + GSH after aging, which might have provided more energy for germination (Fig. 5), hence their germination percentage was almost identical to unaged and non-primed seeds (Fig. 1). These positive results indicated that the enhanced germination of aged oat seeds, due to exogenous ASC and GSH, was likely dependent on the improvement of mitochondrial structures and the activation of mitochondrial antioxidant enzymes.

However, positive effects of ASC were not observed in oat seeds primed before aging. Mitochondrial cristae is poorly developed in both dry and imbibed seeds [1]. However, priming with ASC or ASC + GSH before aging provided favorable conditions for the development of mitochondrial structures and functions. Very similar results were reported in pea (*Pisum sativum*) [40] and soybean (*Glycine max*) [41] seeds. Unfortunately, seed deterioration appears to be localized first within the mitochondria of meristematic cells [3], where it is more likely to cause serious structural and functional mitochondrial damage. We observed that mitochondrial structures deteriorated in embryonic root cells of oat seeds primed with ASC or ASC + GSH before aging (Fig. 2), which might indicate that their mitochondrial membrane systems were difficult to recover during imbibition [25]. Any changes in mitochondrial morphology will be closely associated with the modification of their functions [42]. Compared to aged, non-primed oat seeds, the activities of mitochondrial antioxidant enzymes were not improved in oat seeds primed with ASC or ASC + GSH before aging (Fig. 3). These conditions were unfavorable for removing ROS in the mitochondria of these seeds, hence their mitochondrial H_2_O_2_ and MDA contents also remained a high level (Fig. 4). Additionally, their H_2_O_2_ and MDA contents were higher than those unaged and non-primed (Fig. 4), culminating in the reduced synthesis of many enzymes and restricted ATP production during their imbibition [3, 43]. Thus, the activities of COX and MDH were relatively low in oat seeds primed with ASC or ASC + GSH before aging (Fig. 5), and these seeds could not supply enough energy for germination. The decline in seed germination was most likely due to the degradation of mitochondrial ATP levels in aged soybean [25] and elm [44] seeds. Consequently, the germination percentages of oat seeds primed with ASC or ASC + GSH before aging were not significantly (*P* > 0.05) different from those aged and non-primed, and they were visibly lower than those unaged and non-primed (Fig. 1). These results showed that priming with ASC or ASC + GSH before aging could not prevent the occurrence of seed aging, which was consistent with the outcomes of previous studies [45, 46]. However, compared with oat seeds primed with ASC or ASC + GSH before aging, the activities of mitochondrial SOD, APX, DHAR and GR were enhanced in those primed with GSH before aging (Fig. 3). Additionally, their mitochondrial H_2_O_2_ and MDA contents were reduced (Fig. 4), hence the activities of COX and MDH in these seeds were also improved (Fig. 5). GSH is reported to recycle potential ASC via the ASC-GSH cycle (Fig. 6) [39, 47, 48], but this process might take a long time to complete and its effect might also be relatively weak. Mitochondrial structures and functions might therefore also be relatively slow to develop, which might cause mild structural and functional damage for the mitochondria during seed aging [3].

In our study, mitochondrial DHAR and GR activities in oat seeds primed with GSH or ASC + GSH after aging were markedly higher than those primed with ASC after aging (Fig. 3). This indicated that the ASC regeneration maintained a relatively strong level in mitochondria by post-priming with GSH or ASC + GSH, thereby regulating the balance of redox in the mitochondrial matrix via maintaining ASC in a reduced state (Fig. 6) [39]. This was in agreement with previous studies at the cellular level [38, 49]. However, mitochondrial SOD, APX, and MDHAR activities of oat seeds primed with GSH after aging were markedly lower than those primed with ASC or ASC + GSH after aging (Fig. 3), and the mitochondrial H_2_O_2_ and MDA contents in these seeds were higher (Fig. 4). These results suggested that the antioxidant capacity of oat seeds primed with GSH after aging was less than those primed with ASC or ASC + GSH after aging, hence their lipid peroxidation was enhanced. Consequently, mitochondrial COX and MDH activities of oat seeds primed with GSH after aging were also markedly lower than those primed with ASC or ASC + GSH after aging (Fig. 5), and their germination percentage was also relatively low (Fig. 1). Therefore, these results suggested that the effects of ASC priming were superior to GSH priming for the recovery of aging damage in oat seeds. Previous studies at the cellular level had also demonstrated that the presence of ASC might be crucial for ensuring seed germination [12]. Unexpectedly, the mitochondrial H_2_O_2_ and MDA contents showed no significant (*P* > 0.05) differences between oat seeds primed with ASC and ASC + GSH after aging (Fig. 4), symbolizing a similar level of lipid peroxidation. Thereby, their mitochondrial COX and MDH activities were also not significantly (P > 0.05) different (Fig. 5), showing that they provided similar level of energy for the germination of aged oat seeds, and their germination ability was also not significantly (P > 0.05) different (Fig. 1). These results might imply that positive effects of priming with ASC + GSH after aging were not superior to post-priming with ASC after aging on injured renovation of aged oat seeds. In other words, the regeneration of ASC was induced by post-priming with GSH in oat seeds, but this effect was not obvious under sufficient ASC conditions.

## Conclusions

Our results suggested that pre-priming with ASC, GSH, or ASC + GSH could not inhibit the aging damage in oat seeds. Fortunately, post-priming with ASC, GSH, or ASC + GSH could effectively repair aging damage in oat seeds, and the effect of ASC priming was greater than GSH priming. However, positive effects of priming with ASC + GSH after aging were not greater than priming with ASC after aging on repairing aging damage of oat seeds.

## Methods

### Seed samples

Oat seeds were collected by the Forage Seed Laboratory of China Agricultural University in 2009 and stored in plastic bags at − 20 °C. The original materials were imported from DYCK Forage & Grasses LTD of Canada by Beijing Houderui Trading Co., LTD. At the start of the experiment in December 2014, the moisture content of the seeds was 9.8% (on a fresh-weight basis), the seed germination percentage was 89%, and the oil content of the seeds was 5.0%.

### Seed aging and priming treatments

Seed aging treatments: Primed or non-primed oat seeds with 10% moisture content on a fresh-weight basis (45% relative humidity, RH) were immediately sealed in an aluminum foil bag (0.12 × 0.17 m^2^) and aged for 20 days at 45 °C in a water bath.

Seed priming treatments: Oat seeds with 10% moisture content on a fresh-weight basis (45% RH) were soaked in a solution of ASC, GSH, or ASC + GSH at 20 °C for 0.5 h (selected from 0, 0.5, 1, 3, 6, and 12 h) before or after aging. The concentration of these solutions was 1 mmol L^− 1^ (selected from 0, 0.25, 0.5, 1, 2, 4, and 8 mmol L^− 1^). Thereafter, these seeds were rinsed twice with de-ionized water and air-dried in the dark for 3 days at 25 °C and 45% RH (moisture content reached approximately 10% on a fresh-weight basis).

Each treatment had four replicates, and 800 oat seeds (approximately 20 g) were used for each replicate.

Control 1 (C1) was comprised of unaged, non-primed seeds (approximately 10% moisture content on a fresh-weight basis). Control 2 (C2) was comprised of aged, non-primed seeds (approximately 10% moisture content on a fresh-weight basis).

### Germination tests

Germination tests were conducted according to International Seed Testing Association rules [50]. Four replicates of 100 seeds from each sample were placed into Petri dishes lined with three filter papers wetted with 10 mL de-ionized water, and placed in a growth chamber under a constant temperature of 20 °C. Germination was checked daily for 10 days. On day 10, the final number of normal seedlings was counted, and the length of seedlings was measured.

### Ultrastructural observations of mitochondria in embryonic root cells

Seed samples were selected randomly after different treatments. Embryonic roots, after imbibition for 4 h and without radicle protrusion, were removed and fixed in a 4% glutaraldehyde solution for 24 h and then placed in a refrigerator at 4 °C. The samples of embryonic roots were processed by rinsing with 50 mmol L^− 1^ sodium phosphate buffer, fixing with osmium tetroxide, rinsing with buffer again, dehydrating with an alcohol gradient, and embedding in epoxy resin. After, ultrathin slices were obtained using an LKB8800 III ultramicrotome. Sections were placed on copper grids and stained with 2% aqueous uranyl acetate for 20 min followed by a lead electron staining solution (Katayama, Osaka) for 5 min. Twenty results per treatment were observed by the transmission electron microscopy (Hitachi H-7500).

### Isolation of mitochondria

Mitochondrial extraction was carried out by the method of Yin et al. [51]. The specific extract operation was performed as previously described [7]. Part of the enzyme activity that was detected might be due to minimal microsomal contamination from this method.

### Enzyme assays

SOD (EC 1.15.1.1) activity was determined according to Rao and Sresty [42]. APX (EC 1.11.1.11) activity was assayed according to the method of Nakano and Asada [52]. MDHAR (EC1.6.5.4) activity was measured according to Arrigoni et al. [53]. DHAR (EC 1.8.5.1) activity was assayed according to Dalton et al. [54]. GR (EC 1.6.4.2) activity was assayed according to Madamanchi and Alscher [55]. The detailed determination method of SOD, APX, MDHAR, DHAR and GR was also performed as previously described [7]. COX (EC 1.9.3.1) activity was assayed according to Neuburger et al. [56], and was determined as a decrease in absorbance at 550 nm (ε_550_ = 13.5 mmol^− 1^ cm^− 1^) and 25 °C due to cytochrome c oxidation. MDH (EC 1.1.1.37) activity was measured by monitoring the increase in absorbance at 340 nm (ε_340_ = 6.2 mmol^− 1^ cm^− 1^) and 25 °C due to NADH production according to the methods described by Glatthaar et al. [57]. Reaction systems (1 mL) were mixed with 10 μL mitochondria (20 μg mitochondrial protein) in all enzymes assays.

### The H_2_O_2_ and malondialdehyde (MDA) contents

The H_2_O_2_ content of isolated mitochondria was measured by the method of Patterson et al. [58]. The determination was depened on the changes in absorbance values of the titanium peroxide complex at 415 nm, which was calculated from a standard curve of known H_2_O_2_ concentrations.

MDA content of isolated mitochondria was measured by the method of Bailly et al. [59], which was calculated by the determining absorbance values at 532 and 600 nm. 100 μL isolated mitochondria were mixed into 3 mL reaction systems, which contained 20% (w/v) trichloroacetic acid and 0.5% (w/v) 2-thiobarbituric acid.

### Statistical analyses

Comparisons of mean differences in different treatments were carried out through an analysis of variance (ANOVA), using SPSS for Windows version 13.0. Duncan’s multiple range test (*P* = 0.05) was used to compare the treatment means of physiological indicators for antioxidant systems and lipid peroxidation.

## Data Availability

The datasets used and/or analysed during the current study available from the corresponding author on reasonable request.

## Abbreviations

APX: Ascorbate peroxidase
ASC: Ascorbate acid
ATP: Adenosine triphosphate
COX: Cytochrome c oxidase
DHAR: Dehydroascorbate reductase
GR: Glutathione reductase
GSH: Glutathione
MDA: Malondialdehyde
MDH: Malate dehydrogenase
MDHAR: Monodehydroascorbate reductase;
RH: Relative humidity
ROS: Reactive oxygen species
SOD: Superoxide dismutase.
